# High Morphine Use Disorder Susceptibility Is Predicted by Impaired Learning Ability in Mice

**DOI:** 10.3390/brainsci12121650

**Published:** 2022-12-01

**Authors:** Xue-Fei Hou, Ya-Bo Zhao, Yue-Xiong Yang, Chen Ma, Meng Li, Xin Li, Guo-Rui Ma, Li-Su Zhu, Lin Xu, Qi-Xin Zhou

**Affiliations:** 1 School of Life Sciences, Yunnan University, Kunming 650504, China; 2 Biomedical Engineering Research Center, Kunming Medical University, Kunming 650500, China; 3 Laboratory of Learning and Memory, Key Laboratory of Animal Models and Human Disease Mechanisms, Kunming Institute of Zoology, Kunming 650223, China

**Keywords:** substance uses disorders, morphine, learning ability, FVB mice

## Abstract

An obvious reason for substance uses disorders (SUDs) is drug craving and seeking behavior induced by conditioned context, which is an abnormal solid context memory. The relationship between susceptibility to SUD and learning ability remains unclear in humans and animal models. In this study, we found that susceptibility to morphine use disorder (MUD) was negatively correlated with learning ability in conditioned place preference (CPP) in C57 mice. By using behavioral tests, we identified the FVB mouse as learning impaired. In addition, we discovered that learning-relevant proteins, such as the glutamate receptor subunits GluA1, NR1, and NR2A, were decreased in FVB mice. Finally, we assessed the context learning ability of FVB mice using the CPP test and priming. We found that FVB mice had lower learning performance with respect to normal memory but higher performance of morphine-reinstatement memory. Compared to C57 mice, FVB mice are highly sensitive to MUDs. Our results suggest that SUD susceptibility is predicted by impaired learning ability in mice; therefore, learning ability can play a simple and practical role in identifying high-risk SUD groups.

## 1. Introduction

The World Drug Report released by the United Nations Office on Drugs and Crime notes that approximately 5.5% of the global 15 to 64-year-old population had used drugs at least once in the past year, and 13% of them suffered from drug use disorders. This shows that the risk of drug susceptibility in the population varies among individuals. According to the report, individuals with poor educational attainment account for more than 50% of people with SUD; thus, a low education level is an obvious risk factor for SUD. A study including 380 students found that susceptibility to SUD can be predicted based on the cognitive dimension [1]. Children with lower IQ and lower levels of education before smoking onset are more likely to develop smoking use disorder later in life [2]. The brain regions involved in cognitive processes, such as executive function, attention, reward, and memory function, differ between SUD and non-SUD population studies [3,4,5,6]. The above studies show that there is a certain degree of correlation between cognition and SUD. SUD high-risk groups cannot currently be identified with a reliable tool. Therefore, it is of paramount significance to explore the use of learning ability to identify high-risk groups of SUD and develop simple and easy identification tools for drug management and addiction treatment.

Learning is an ability necessary for survival, and animal models are usually used to study learning. In rodents, behavioral paradigms such as novel environmental recognition, socialization, and the water maze can be used to study memory formation, renewal, and extinction [7]. Drug-related memory is an obvious cause of SUD, preclinical models of SUD are essential for elucidating the neurobiological mechanisms that contribute to drug-related behaviors. Currently, the formation, reactivation and extinction of drug memory are thought to be related to associative memory [8].

The CPP model is a well-established behavioral paradigm for studying SUD, particularly for measuring the association formed between drugs and the contextual environment [9]. One of the most significant components of SUD is the associative learning process; thus, drug cravings and compulsive-seeking behaviors triggered by drug-related contextual memory are often examined to study SUD. In the conditioning stage of drug use, a strong learned associations is formed between drug-associated environmental cues and drug effects [6]. The formation of this reinforcement memory (acquisition effects) is typically assessed by an increase in CPP test scores, which becomes the basis for drug craving and drug-seeking after SUD. After CPP formation, animals undergo extinction training, which involves exposing animals to a previous drug-paired environment (retrieval and renewal) and no drug use (new condition), so extinction is considered novel learning. Clinical extinction procedures can suppress conditioned responses to drug cues [10]. Following conditioned memory formation or extinction, re-exposure to the drug causes reinstatement of the conditioned response. Reinstatement effects usually manifest as an increase in CPP scores following priming [11,12]. CPP priming is used to model drug seeking and relapse [9,11,12,13], a defining characteristic of SUD. Therefore, increases in CPP scores triggered by the environment and drugs are often used as a measure of SUD susceptibility. Based on our previous study of CPP and morphine’s pharmacological properties [14,15,16,17,18], 4-day morphine training was primarily used to induce an association between drug and condition contexts. Several studies have demonstrated that repeated morphine training for 9 or 12 days leads to severe withdrawal syndromes in mice [16,17], and 12-day morphine training was used to study conditioning and reinstatement in mice with substance abuse disorders.

Studies have shown that SUD and learning ability may share a common neural basis [19,20]. Glutamate is a neurotransmitter that is widely distributed in the prefrontal cortex, hippocampus, and thalamus and contributes to the neural plasticity underlying learning and memory [21]. It plays an instrumental role in SUD, particularly regarding relapse behaviors [22,23]. α-Amino-3-hydroxy-5-methyl-4-isoxazolepropionic acid (AMPA) and N-methyl-D-aspartate receptor (NMDA) glutamate receptors (AMPARs/NMDARs) are the predominant receptors in neurons. NMDA, NR2A, and AMPAR GluA1 play a role in long-term potentiation (LTP) [24,25,26], which is correlated with learning and memory, and learning is impaired when NR2A is absent [27]. Hippocampal glutamate synaptic plasticity and spatial cognitive function were significantly impaired in animals treated chronically with morphine [28]. In addition, the hippocampus is critical for extinction and relapse prevention [29,30], and morphine-induced conditioned place preference (CPP) significantly increases dendritic spine density in CA1 and the dentate gyrus, which are significantly correlated with CPP scores [31].

In this study, a mouse learning ability assessment method and a CPP model were established to test whether poor learning ability is associated with SUD susceptibility, which may be useful in identifying high-risk groups in the future.

## 2. Methods

### 2.1. Animals

Adult male C57BL/6J mice (from the Animal Center of Kunming Medical University, 12 weeks of age, weighing 18–22 g) and FVB/NJ mice (from Ding Yu-Quang’s research group, 12 weeks of age, weighing 18–22 g) were used. Animals were housed four per cage and given free access to food and water under a 12 h light/dark cycle in a temperature-regulated environment maintained at 22 ± 1 °C at the Kunming Institute of Zoology. The experimental procedures were approved by the Animal Ethics Committee of the Kunming Institute of Zoology (No. SMKX-SQ-20200803-132).

### 2.2. CPP Behavioral Experiment

The apparatus for the CPP paradigm was similar to that used by Carr and White [32]. The CPP box consists of two same-sized chambers (20 × 20 × 20 cm) and a connecting channel (10 × 20 × 20 cm). The chamber has different horizontal and vertical stripes as visual cues, and there are switchable doors on both sides of the passage. CPP consisted of three distinct phases: preconditioning, conditioning, and postconditioning. Postconditioning includes post training testing, extinction, and priming.

In the CPP habituation stage (preconditioning), the mice were allowed to freely explore the two chambers for 15 min from days 1 to 3. Noldus software was used to track and record the time the mice spent in each box every day. Mice that spent more than 600 s or less than 300 s in one chamber over all three days were removed from subsequent CPP experiments. Mice meeting the criteria were randomly assigned to three groups during training: a 4-day training group (days 4–7), a 12-day training group (days 4–15), and a control group (In addition to changing morphine into saline, others were consistent with the training group).

During the CPP training stage (conditioning), the 4-day training group consisted of a total of eight training sessions (four saline and four morphine), and the 12-day group consisted of 24 sessions (12 saline and 12 morphine). These sessions were conducted twice each day with a 6 h interval between [33,34]. On each of these days, the mice received two conditioning sessions with morphine (20 mg/kg, subcutaneous injection) [33] in the morning and with saline (10 mL/kg, subcutaneous injection) in the afternoon. During these sessions, the mice were confined to the box by closing the door. The mice were injected with morphine and then put into one of the chambers (set as the morphine-paired side) for a 30 min morphine training session. They were then returned to their cage for 6 h. The mice were then injected with saline and placed on the other side of the box (designated the paired saline side) for 30 min for a saline training session. After the session, the mice were returned to their home cage until the next day.

During the test (post-conditioning) phase, on the 8th day (4-day training group) or the 15th day (12-day training group), the mice were free to explore both chambers for 15 min. The time that mice stayed on the morphine-paired side was used as the test CPP score, which is a method to assess CPP learning ability. During the priming (post-conditioning) phase, on the 15th day (4-day training group) or the 23rd day (12-day training group), the mice were given a small dose of morphine (5 mg/kg, subcutaneous) for priming and allowed to explore the two boxes for 15 min. The time that the mice stayed on the morphine-paired side was used as the priming CPP score, which was an evaluation method for SUD susceptibility.

During the training, testing, and priming phases, the control group was treated the same as the training group except that morphine was replaced with saline.

The extinction phase was scheduled from the 8th to 12th days after the completion of the 4-day morphine training [10,35]. The extinction phase included eight sessions (four morning tests and four afternoon extinctions), which were conducted twice a day with a 2 h interval between [10]. The mice were divided into a saline–saline extinction experiment (saline extinction) and a saline extinction experiment (morphine priming in the morning) in the extinction phase. On each of these days, the mice were injected with saline (in the saline extinction experiment, 2.5 mL/kg) or morphine (in the saline extinction experiment, 5 mg/kg) subcutaneously followed by placing them in the CPP box (with the channel door open) for free exploration for 15 min in the morning. In the afternoon, the mice were injected with saline (2.5 mL/kg) subcutaneously and put into the box of the morphine-paired chamber (with the channel door closed) for 30 min for saline extinction. After the session, the mice were returned to their home cage until the next day.

The priming, test, and extinction CPP scores were calculated as the time that the mice spent exploring the morphine paired side chamber minus the ipsilateral time of exploration in the adaptation period in day3.

### 2.3. Open Field Test (OF)

Mice were placed in a 50 × 50 × 40 cm cage with a white background. Locomotor activity and time in the center quadrant were monitored using AnyMaze (Stoelting LTD, Vision 4.9.9, USA) software for 30 min.

### 2.4. Morris Water Maze (MWM)

Mice were placed in a circular water tank (150 cm in diameter and 50 cm in height) filled with water to a depth of 20 cm at a temperature of 20 ± 1 °C. The water was dyed with a soluble white dye. The pool displayed distinctive marks that served as visual cues. The AnyMaze (Stoelting Europe LTD., Vision 4.9.9) automatic tracking system software was used to record the latency and crossing number. The Morris water maze process consisted of the following steps: the mice were adapted to the water maze on day 1 for 90 s with a platform visible and then trained for 4 days with 4 trials for 60 s per day using a platform underwater. Performance was measured by timing the escape latencies during each training trial. Finally, the mice were subjected to a probe test for 60 s on day 6 without the platform, and the number of platform location crossings was measured.

### 2.5. Contextual Fear Conditioning (CFC)

The mice were placed in a training box (MED Associates, USA) that they were allowed to freely explore for 150 s and then began foot shock training (five trials, 0.8 mA, 2 s) (session of shock stimulus interval (s): 150, 90, 80, 100, 70) at various intervals. After training for 30 min, 1 day, and 7 days, the mice were placed back in the box without foot shocks for 10 min. The freezing time was automatically quantified using software (Med Associates, Inc, VideoFreeze software, USA) and recorded to represent the level of fear learning and memory.

### 2.6. Social Preference (SP) and Novel Object Recognition (NOR)

The social preference testing apparatus consisted of a rectangular box (50 × 30 × 15 cm). The center chamber was smaller (10 × 30 cm) than the other two chambers, which were of equal size (20 × 30 cm). The mice were placed in a three-chamber apparatus for three 10 min sessions. In session 1, the test mouse was first placed in the middle chamber and allowed to freely explore the two-sided chambers. In session 2, stranger mouse no. 1 was enclosed in a wire cup placed in a one-sided chamber, and an empty wire cup was placed in the other side of the chamber. The test mouse was permitted to explore stranger mouse no. 1 (stranger mouse) and an empty wire cup (novel object). In session 3, stranger mouse no. 2 was enclosed in the empty wire cup and placed in the same location as in session 2. The test mouse was permitted to explore stranger mouse no. 2 (novel mouse) and familiar mouse no. 1 (familiar mouse). The time spent sniffing the mouse and the empty cup was recorded using AnyMaze (Stoelting Europe LTD, Vision 4.9.9) software. The chambers and wire cups were cleaned with 70% ethanol between sessions and after each test.

The novel object recognition tests included two 10 min sessions. The mouse explored the side chambers with two identical objects in the first session. After 10 min back in the home cage, the mouse reexplored the chambers with an original object (familiar) and a novel object that were at the same location as that in the second session. AnyMaze (Stoelting Europe LTD, Vision 4.9.9) software was used to record the times that the mice were spent actively sniffing the novel and original objects.

### 2.7. Tail Flick Latency

A thermal stimulus (laser) was applied to the animal’s tail, provoking withdrawal of the tail (Panlab LE7106). The time of withdrawal movement was recorded as tail flick latency (40 W, 20 s tail radiation heating). Tail flick latencies were determined before and 30 min after the injection of morphine. To prevent any burn injury to the mice, a maximum time of 20 s was used in the experiments.

### 2.8. Western Blotting

After the behavioral experiment, the mouse brain was quickly removed, and hippocampal tissues (six per group) were placed in ice-cold saline. The tissues were homogenized with RIPA buffer and protease inhibitors for 10 min and then centrifuged at 12,000 rpm for 30 min at 4 °C. The supernatants of the samples were assayed for protein content, diluted with 1:4 sample buffer and heated at 95 °C for 5 min. The proteins were loaded onto 10% sodium dodecyl sulfate (SDS) polyacrylamide gels and then transferred onto PVDF membranes. The membranes were incubated with blocking buffer (5% nonfat milk and 0.1% Tween-20 in Tris-buffered saline [TBST]) for 1 h at 27 °C. The membranes were incubated with the following primary antibodies overnight at 4 °C: anti-GluA1 and anti-GluA3 (Abcam 1:2000), anti-GluA2 (Millipore 1:2000), anti-NR1 (Sigma 1:1000), anti-NR2A and anti-NR2B (Abcam 1:2000), anti-ERK (Abcam 1:1000), anti-CREB (Abcam 1:1000), and anti-PSD95 (CST 1:1000). After three washes with TBST, the membranes were incubated with IRDye 700DW- or 800DW-conjugated anti-mouse or anti-rabbit IgG (1:10,000) for 1 h at room temperature, washed with PBS, and scanned to detect fluorescence with the LI-COR Odyssey detection system.

The investigators were blinded to the group assignment of the animals in all of the aforementioned experiments.

### 2.9. Statistical Analyses

The statistics were analyzed using GraphPad Prism (version 9.2.0). Numerical data are presented as the mean ± SEM. Statistical significance was analyzed using Student’s *t*-test for two groups and analysis of variance (ANOVA) for three or more groups. One-way ANOVA and two-way ANOVA with post hoc tests (Fisher’s LSD test) were used to analyze differences in the learning curves of behavioral paradigms. A chi-square test was used to analyze the ratio between the two groups. For all analyses, *p* < 0.05 was considered statistically significant.

## 3. Results

### 3.1. CPP Box Spontaneous Activity Score in C57 Mice

**Figure 1:**
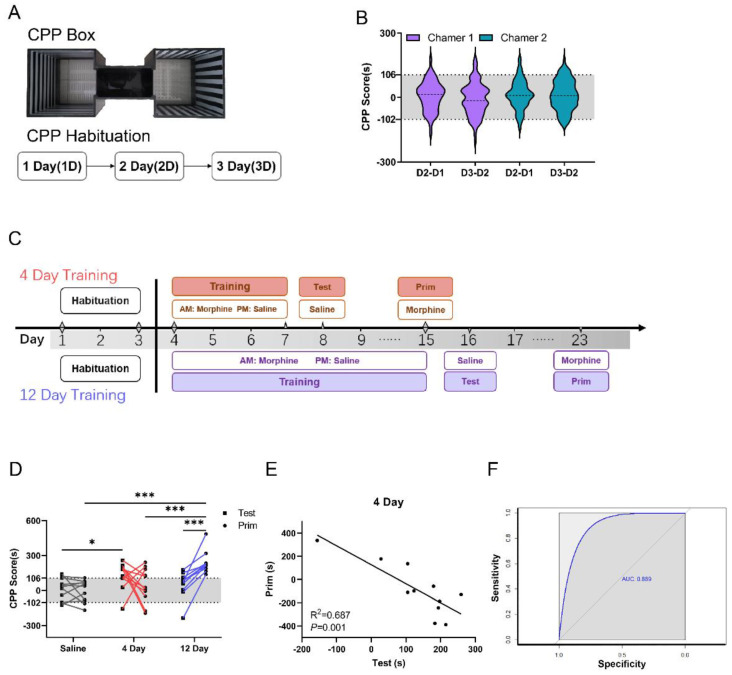
**Negative correlation between test and priming in C57.** (**A**). The CPP box and CPP habitation; the C57 mice inhabited this box for three days. (**B**). The scores in the two chambers are distributed on a scale of 106 to −102. D2−D1: Day 2 minus day 1 in chamber 1 or chamber 2. D3−D2: Day 3 minus day 2 in chamber 1 or chamber 2. Scores were determined by subtracting the time spent on the same side each day. n = 115. (**C**). Experimental Procedures. After 3 days of habituation, C57 mice were trained for 4 days or 12 days with morphine in the morning (AM) and saline in the afternoon (PM). The testing was performed 1 day after morphine training, and the priming was performed 7 days after morphine training. CPP scores were determined by subtracting the times spent on the morphine training side each day. (**D**). The effect of morphine training on the test and priming in C57 mice (n = 11, two-way ANOVA). The dark gray line represents 106 s. (**E**). Correlation between the test and priming (R^2^ = 0.687, *p* < 0.001) showing a negative correlation between the C57 test and priming. (**F**). Receiver operating characteristic (ROC) curves were generated to evaluate the feasibility of using CPP test scores as a diagnostic indicator. * *p* < 0.05, *** *p* <0.001.

We used an unbiased place conditioning paradigm, adopting a CPP box that consisted of two chambers of the same shape and size, with visual cues as horizontal and vertical stripes and tactile cues as circular or elongated holes in the floor (Figure 1A), which is beneficial for mice that do not have an obvious preference for bilateral chambers. The habituation process consisted of 15 min of free access to the boxes for three consecutive days (Figure 1A), which helped mice adapt to the environment of the CPP box. By detecting the spontaneous activity of 115 C57 mice in the CPP box over three habituation days, we found no difference in CPP scores between day 3 (D3-D2) and day 2 (D2-D1) in the same chambers and no difference in CPP scores between the two chambers (Figure 1B, *p* > 0.05, one-way ANOVA). This result indicates that C57 mice show no preference for either chamber during the habituation period. According to the distribution of the data, we found that these data fit the normal distribution (*p* > 0.05, Kolmogorov–Smirnov test). According to the normal value setting method (mean ± 2 SD), we calculated a range of spontaneous activity variation with an upper limit of 106 s and a lower limit of −102 s (Figure 1B, n = 115, gray range). Then, a CPP score of 106 s was used as a discriminant criterion to assess the learning ability of the mice, and scores greater than 106 s were considered to indicate good place preference toward the drug-paired chamber.

### 3.2. Learning Ability Is Negatively Correlated with SUD Susceptibility in C57 Mice

Morphine training groups for C57 CPP were designed for 4 and 12 days. The morphine paired side time was recorded during the CPP test and priming, and the CPP score was calculated (Figure 1C). After 3 days of habituation, the mice in the 4-day training group were trained with morphine (20 mg/kg) and saline alternately for 4 days, tested with saline on the 8th day, and primed with morphine (5 mg/kg) on the 15th day; the mice in the 12-day training group were trained with morphine (20 mg/kg) and saline alternately for 12 days, tested with saline on the 16th day, and primed with morphine (5 mg/kg) on the 23nd day. The CPP scores were used to detect the acquisition effects of associative memory between morphine and the context as an indicator of learning ability [6,34]. The results showed that the CPP scores of the saline group were not significantly different in the testing and priming phases; in the 4-day morphine group, during the test phase, the CPP scores of the mice were higher than those in the saline group. In the 12-day morphine group, during the priming phase, the CPP scores were higher than those in the saline and 4-day morphine groups (Figure 1D; n = 10, F (2,60) =8.959, *p =* 0.02, *Saline: Test* vs. *4 Day: Test, p* < 0.001, Saline: Prim vs. 12 Day: Prim, * *p* < 0.001, 4 Day: Prim vs. 12 Day: Prim, *** *p* < 0.001, 12 Day: Test vs. 12 Day: Prim, two-way ANOVA). These results suggest that 4 days of morphine training can lead to the acquisition of reward-related context. The priming results of the 12-day morphine training showed that training led the mice to form strong reinstatement memories, which represent a higher likelihood of relapse. Further analysis of the 4-day morphine group revealed that some of the mice had CPP scores lower than 106 s in the test phase but higher than 106 s in the priming phase (Figure 1D 4 day). Therefore, we speculate that there may be a relationship between learning in morphine CPP and SUD susceptibility in C57 mice. By analyzing the correlation between the CPP scores in the test and priming phases, we found that there was a linear negative correlation between the two phases in the 4-day morphine training (Figure 1E), which means that there is an inverse relationship between learning ability and SUD susceptibility. To assess the leaning ability for morphine use disorder (MUD) susceptibility, ROC curves were constructed. The area under curve (AUC) of leaning ability to distinguish high MUD susceptibility from low MUD susceptibility was 0.905 (95% CI: 0.772–0.954) with the optical sensitivity and specificity of 82.8% and 81.8%, respectively. The results showed that mice that scored poorly in the test phase were more likely to have high scores in the priming phase. This suggests that mice with poor CPP learning ability may have higher susceptibility to SUD.

### 3.3. Poor Learning Ability in FVB Mice

**Figure 2:**
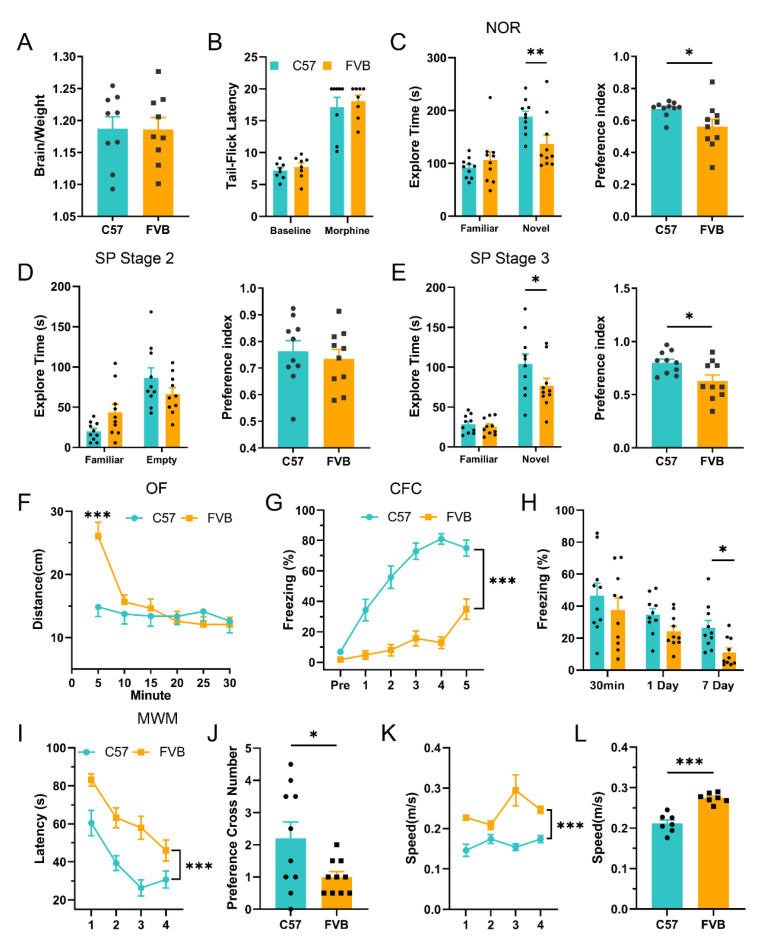
**Normal learning ability was poor in FVB mice.** (**A**). The weight percent ratio of brain to body in C57 and FVB mice (n = 9, unpaired *t*-test). (**B**). The tail flick test in C57 and FVB mice (n = 9, two-way ANOVA), baseline and 30 min after morphine. (**C**). Novel object recognition (NOR) test in C57 and FVB mice shows the time spent with a familiar and a novel object (n = 10, two-way ANOVA, *t*-test). (Left): Exploration time. (Right): Preference index. (**D**). Social preference test (SP) stage 2 shows the exploration time (left) and preference index (right) for a familiar mouse and an empty cage in C57 and FVB mice (n = 10, two-way ANOVA, *t*-test). (**E**). Social preference test stage 3 compares the exploration time (left) and preference index (right) for a novel mouse and familiar mouse in C57 and FVB mice (two-way ANOVA, *t*-test). (**F**). The open field (OF) test collected the distance of motion every five minutes over 30 min between C57 and FVB mice (n = 10, RM two-way ANOVA). (**G**). Learning curve during contextual fear conditioning (CFC) training (n = 10, RM two-way ANOVA). (**H**). Contextual fear conditioning memory test at 30 min, 1 day, and 7 days in C57 and FVB mice (n = 10, two-way ANOVA). (**I**). Learning curve during Morris water maze training (n = 10, two-way ANOVA). (**J**). The platform cross number in the Morris water maze memory test between C57 and FVB mice (n = 10, *t*-test). (**K**). Swimming speed during Morris water maze training (n = 7, two-way ANOVA). (**L**). Swimming speed Morris water maze memory test between C57 and FVB mice (n = 7, *t*-test). * *p* < 0.05, ** *p* < 0.001, *** *p* < 0.001, Mean ± SEM.

We used behavioral methods, including the NOR, SP, MWM, and CFC tests, to detect short-term and long-term memory in different mouse strains. All of these methods are effective ways to evaluate animal learning and memory ability. Brain weight is altered in many cognition-related diseases, such as Alzheimer’s disease (AD) [36]. Thus, we examined changes in brain weight and found no significant differences in FVB mice compared to C57 mice (Figure 2A; n = 9, *p* = 0.968, *t*-test). Because morphine has an analgesic effect, we tested tail flick latency between FVB and C57 and found no significant differences between baseline and morphine analgesic responses (Figure 2B; n = 8, *p* = 0.491, baseline, *p* = 0.602, morphine, *t*-test), indicating that there was no significant difference in the effect of morphine analgesia between the two mouse strains. We next compared learning and memory ability between FVB and C57. Short-term memory was examined by novel object recognition and social preference experiments. In novel object recognition, FVB mice had a lower exploration time and preference index for novel objects than C57 mice (Figure 2C; n = 10, F(1,36) = 6.907, *p* = 0.048, Familiar, ** *p* = 0.006, Novel, two-way ANOVA, * *p* = 0.031, preference index, *t*-test). In the social preference experiment, there was no significant difference between FVB mice and C57 mice in social memory ability (an empty cage on one side; Figure 2D; n = 10, F(1,36) = 6.019, *p* = 0.071, Familiar, *p* = 0.115, Empty, two-way ANOVA, *p* = 0.596, preference index, *t*-test). However, between familiar and unfamiliar mice, FVB mice performed worse than C57 mice in spending time with unfamiliar mice (Figure 2E; n = 10, F(1,36) = 2.206, *p* = 0.796, Familiar, * *p* = 0.023, Novel, two-way ANOVA, * *p* = 0.018, preference index, *t*-test). The NOR and SP behavior results suggest that FVB mice have reduced short-term memory ability compared to C57 mice. In the open field (OF) experiment, FVB mice showed no difference in activity throughout the experimental period but moved significantly longer than C57 mice during the first 5 min (Figure 2F; n = 10, F(1,18) = 1.034, *** *p* < 0.001, 5 min, RM two-way ANOVA), suggesting that the anxiety state of the two mice was not significantly different. Next, we used the MWM and CFC to compare long-term memory in mice. During the training and test phases, FVB mice exhibited poorer learning performance and retrieval performance, whereas the differences in the learning phase were more pronounced (Figure 2G; I, n = 10, F(1,18) = 111.4, *** *p* < 0.001, CFC, F(1,18) = 26.30, *** *p* < 0.001, MWM, RM two-way ANVOA) and memory retrieval phase (Figure 2H; J, n = 10, F(2,54) = 0.197, *p* = 0.230, 30 min, *p* = 0.166, 1 Day, * *p* = 0.044, 7 Day, CFC, two-way ANOVA, * *p* = 0.037, MWM, *t*-test). At the same time, FVB mice swam faster than C57 mice(Figure 2 K, n = 7, F(1,12) = 44.87, *** *p* < 0.001, RM two-way ANVOA; Figure 2L, *** *p* < 0.001, *t*-test). These results suggest that FVB mice have deficits in long-term memory formation and retrieval. In summary, these results confirmed that the normal learning and memory abilities of FVB mice were significantly lower than those of C57 mice.

**Figure 3:**
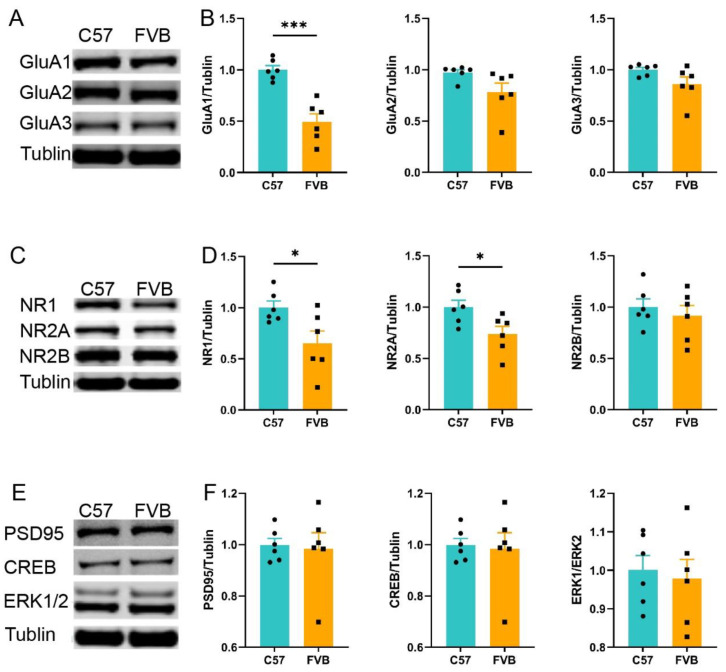
**FVB mice showed a decrease in AMPAS and NMDARs compared with C57 mice**. (**A**). Western blot analysis of AMPAs in hippocampal protein content between C57 and FVB mice. (**B**). Relative protein content level of AMPAs (**left**: GluA1, **middle**: GluA2, **right**: GluA3; n = 6, *t*-test). (**C**). Western blot analysis of NMDARs in hippocampal protein content between C57 and FVB mice. (**D**). Relative protein content level of NMDARs (**left**: NR1, **middle**: NR2A, **right**: NR2B; n = 6; *t*-test). (**E**). Western blot analysis of other protein contents in the hippocampus between C57 and FVB mice (n = 6). (**F**). Relative protein content levels of ERK (**left**), CREB (**middle**), and PSD95 (**right**). * *p* < 0.05, *** *p* < 0.001, Mean ± SEM.

To further test the learning ability of mice, we selected proteins related to learning and memory for detection. AMPARs and NMDARs are key components of the glutamate system, which is associated with learning and memory. Hippocampal GluA1 in FVB mice was particularly reduced (Figure 3A,B, n = 10, *** *p* < 0.001, GluA1, *p* = 0.065, GluA2, *p* = 0.084, GluA3, *t*-test), and the reduction in NR1 and NR2A was also evident in FVB mice (Figure 3C,D, n = 10 * *p* = 0.028, NR1, * *p* = 0.026, NR2A, *p* = 0.522, NR2B, *t*-test), while other learning-related proteins were not significantly different (Figure 3E,F; n = 10, *p* = 0.731, PSD95, *p* = 0.834, CREB, *p* = 0.461, ERK, *t*-test)(). These results indicated that there were differences in glutamate receptor abundance between the two mouse strains, and FVB mice showed lower expression of learning- and memory-related proteins.

In conclusion, both behavioral and glutamate protein results indicated that the normal learning ability of FVB mice was worse than that of C57 mice.

### 3.4. FVB Mice Have Higher SUD Susceptibility

**Figure 4:**
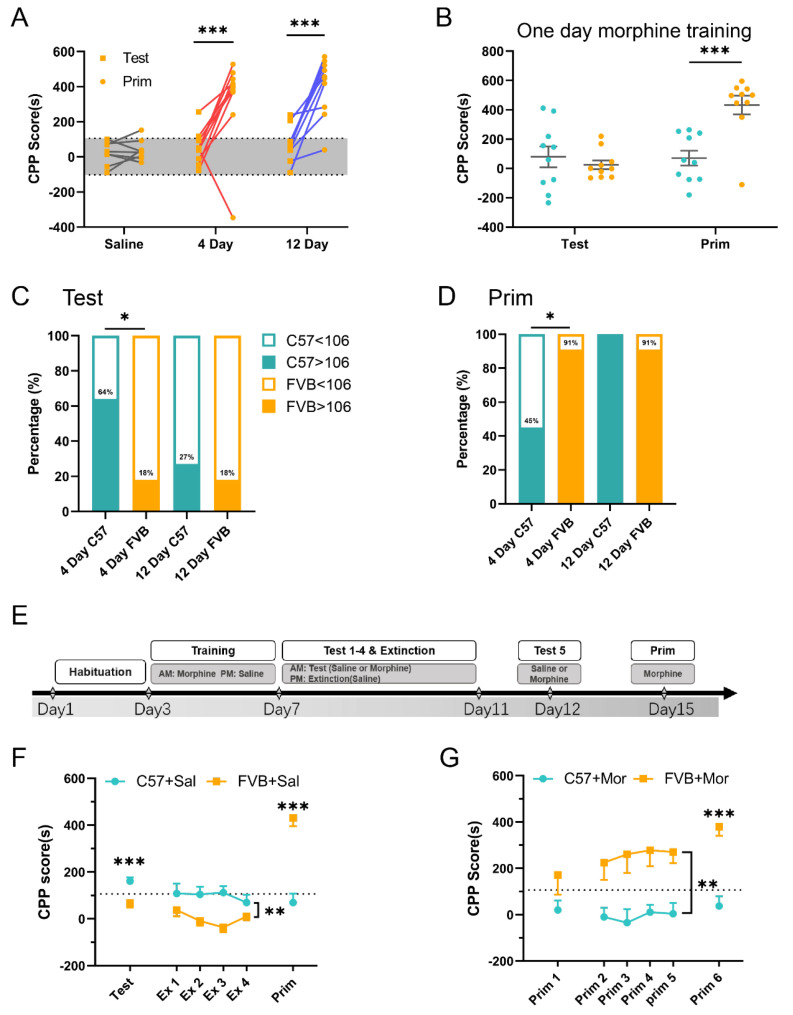
**Relationship between weak learning ability and strong SUD susceptibility in FVB compared with C57 mice.** (**A**). The effect of morphine training on the test and priming in FVB mice (n = 11, two-way ANOVA). Gray area range: 106 to −102. (**B**). Comparison of the test and priming performance after morphine training for one day in C57 and FVB mice (n = 10, two-way ANOVA). (**C**,**D**). The percentage of CPP scores greater and less than 106 in the test and priming for morphine training for 4 days and 12 days between C57 and FVB mice (n = 11, chi-square test). (**E**). Experimental scheme. After 3 days of habitation, C57 mice and FVB mice underwent CPP training with morphine in the morning (am) and saline in the afternoon (pm) for 4 days and then underwent extinction trials for 4 days (for saline extinction, am: saline, pm: saline, (**F**); for morphine priming, am: morphine, pm: saline, (**G**)). Low-dose morphine was then administered 3 days after extinction. CPP scores were determined by subtracting the times spent on the morphine training side each day. (**F**). The effect of saline on the extinction trials in C57 and FVB mice (n = 10, RM two-way ANOVA, *t*-test). The black line represents 106 s. (**G**). The effect of priming on saline extinction trials (n = 10, RM two-way ANOVA, *t*-test). * *p* < 0.05, ** *p* < 0.001, *** *p* < 0.001, Mean ± SEM.

In the CPP experiment on C57 mice, we found a negative correlation between learning ability and SUD susceptibility, i.e., mice with poor learning ability showed high SUD susceptibility. To validate this hypothesis, we sought to understand whether FVB mice had heightened SUD susceptibility. Using the CPP experimental protocol, we found that the CPP scores of the FVB mice in the 4-day and 12-day morphine training groups increased significantly in the priming phase (Figure 4A; n = 10, F(2,50) = 5.893, *p* = 0.799, Saline, *** *p* < 0.001, 4 Day, *** *p* < 0.001, 12 Day, two-way ANOVA). This proof supported the hypothesis that FVB mice with poor learning ability show high SUD susceptibility. We analyzed the ratio (no. >106/no. total; no. <106/no. total) of C57 mice and FVB mice 24 h after 4 and 12 days of morphine training and found that in the morphine training groups, C57 mice performed significantly better learning ability than FVB mice in the testing phase (Figure 4C; n = 11, * *p* = 0.030, Test: 4 Day, *p* = 0.610, Test: 12 Day, two-sided chi-square test), and C57 mice performed significantly worse than FVB mice in the priming phase (Figure 4D; n = 11, * *p* = 0.022, Prim: 4 Day, *p* = 0.306, Prim: 12 Day, two-sided chi-square test). This proof further confirmed our hypothesis in C57 subjects. We used 1-day morphine training, and the results also showed a significant increase in CPP scores of FVB mice during the priming phase compared with C57 mice (Figure 4B; n = 10, F(1,36) = 13.58, *p* = 0.504, Test, *** *p* < 0.001, Prim, two-way ANOVA). The results suggested that FVB mice had strong SUD susceptibility. We then performed an extinction experiment for 4 days after training to further investigate whether strong morphine memory in FVB mice could be extinguished. First, the extinction experiment was performed with saline in the saline extinction experiment protocol in C57 and FVB mice (Figure 4E). Compared to FVB mice, the CPP scores of the C57 mice decreased significantly in the saline extinction stage, but the scores of the FVB mice in the priming phase were significantly higher than those of the C57 mice (Figure 4F; n = 10, F(3,54) = 3.06, * *p* = 0.035, extinction 1–4, two-way ANOVA, *** *p* < 0.001, test, *** *p* < 0.001, Prim, *t*-test). This result suggests that morphine-free conditioning memory and morphine-triggered memory exist in CPP training, in which morphine-free memory can be extinguished slowly over time; however, morphine-triggered memory is strongly present and cannot be extinguished. To confirm that morphine-triggered memory cannot be extinguished, we adopted the morphine extinction protocol in C57 and FVB mice; the results showed that the FVB mice still had higher CPP scores than the C57 mice during the extinction and priming phases (Figure 4G; n = 10, F(3,54) = 0.215, *p* = 0.885, extinction 1–4, two-way ANOVA, *p* = 0.125, *t*-test, *** *p* < 0.001, Prim, *t*-test). This result suggests that reinstatement memory in FVB mice is a form of strong memory that cannot be decreased. We found that FVB mice with poor learning and memory ability achieved better reinstatement outcomes. However, reinstated learning and memory cannot be eliminated by the current extinction method. Considering that reinstatement is an evaluation index of SUD relapse, the results of this experiment demonstrate that FVB mice with poor learning ability have high SUD susceptibility.

## 4. Discussion

In this study, we found that there is a negative correlation between learning ability and SUD susceptibility, indicating that mice with poor learning ability are more susceptible to SUD. FVB mice show poor learning and memory ability in short- and long-term learning tasks and exhibit a decrease in glutamate receptors GluA1, NR1 and NR2A in the hippocampus. We observed that FVB mice exhibited lower morphine learning memory (in the test phase) and higher morphine reinstatement memory (in the priming phase) in morphine CPP and exhibited higher reinstatement capacity even when induced with only a single dose of morphine. In other words, FVB with poor learning ability and low expression of proteins related to learning ability are more susceptible to SUD. In summary, our results suggest an increased susceptibility to morphine use disorder with impaired learning ability, which may be related to alterations in glutamate receptor subunits.

Using statistical methods, we found that there should be a time range for spontaneous activity in mice during CPP habituation. The CPP paradigm includes many types of SUD information, such as memory, motivation, and relapse [9,13]. In this study, the experimental box was designed with horizontal and vertical stripes to create an unbiased CPP model [9,13,37]. However, previous studies have focused more on the behavior of animals in CPP after drug effects. In the process of establishing the unbiased CPP model in this study, we focused on the spontaneous activity of mice, which is not common in previous studies. We calculated the normal range of spontaneous activity based on data and statistical methods. The calculated range was used as a criterion to evaluate the condition learning ability of mice and further explored the negative correlation between the testing and the priming of morphine use disorder in C57 mice. The range of spontaneous activity is also obtained with the existing equipment and conditions in the laboratory, but there are some variations in different laboratory conditions. Therefore, the value range is a reference when the laboratory conditions are considered.

The hypothesis that reduced learning and memory may predict higher levels of SUD susceptibility is strongly supported by our results. Humans and animals have different susceptibilities to drugs. A high percentage of the population is exposed to addictive substances due to diseases such as pain and cough. However, only a small number are at elevated risk of SUD [38,39]. According to a World Health Organization survey, approximately 275 million people used addictive substances in 2020. Approximately 13% of drug users will become addicted to drugs, which also indicates that the susceptibility to SUD varies across the population. In our study, we found that some C57 mice showed increased CPP scores during four days of training. However, the proportion of C57 mouse priming was significantly lower than that of FVB mice, which indicated that SUD susceptibility varied across individual mice and strains. The characteristics of this individual difference are consistent with the performance of the population. Low IQ testing and low education levels indicate that it is easier for children to become addicted to nicotine and that addiction will persist for longer [40,41]. There is some correlation between intelligence and SUD, but this relationship has not been confirmed in animal models. In this CPP experiment in mice, we found that learning ability and SUD susceptibility were negatively correlated; that is, mice with worse learning ability have higher SUD susceptibility. This hypothesis was further supported by work on FVB mice, which exhibit deficiencies in learning in memory. Drug relapse involves learned associations between drug-associated environmental cues and drug effects [10]. The extinction method is typically used to reduce drug cravings and relapse during abstinence [10,11,35]. Our extinction results show that the CPP scores of C57 mice can be decreased (Figure 4F ex1-4). In other words, the persistence of drug-seeking behavior in the absence of the drug (drug-free state) can be extinct. FVB has a low CPP score, and the extinction effect is not obvious when compared with C57. Reinstatement of response through a priming injection of a drug is acknowledged to be a reliable model for studying the mechanisms involved in drug craving and relapse [9,11,42]. The conditioned place preference induced by opioids can be reinstated by drug priming following acquisition and subsequent extinction [11]. In our study, mice showed significant SUD behavior at priming after extinction (Figure 4F prim) and acquisition (Figure 4G prim1). On the following day and three days after morphine priming, CPP scores were not decreased by morphine priming (Figure 4G prim2-5, 6). These results suggest that the reinstatement of drugs is extremely firm and difficult to eliminate, which is consistent with a previous study [11,43]. We also observed that the testing and priming results were obtained under morphine-free and morphine states, which means that drug-associated environmental cues play an important role in reward-associated memory and SUD susceptibility. However, our results do not completely eliminate the possibility of the special response of FVB animals to morphine [44,45,46].

The brain regions and neural processes involved in SUD and cognition (including learning, memory, and reasoning) functions are widely coincidental [19]. The glutamate system plays a key role in SUD, and glutamate release activates postsynaptic AMPA receptors to generate synaptic transmission. Moreover, NMDA receptors can be activated under certain conditions to generate synaptic plasticity, including LTP and long-term depression (LTD) [47,48]. NR2A and GluA1 play key roles in LTP [17,18,19], and discrimination learning is impaired when NR2A is absent [27]. Social memory integration and associative memory were also inhibited when NMDARs and AMPARs in the medial prefrontal cortex (mPFC) and hippocampus were blocked [49,50]. These results are consistent with our findings that reductions in NR2A and GluA1 are associated with impaired LTP, which is a mechanism underlying impaired learning and memory. Long-term use of ketamine leads to cognitive impairment accompanied by a decrease in the expression of GluA1 and NR1 in the hippocampus [51,52], and cocaine leads to GluA1 production and transport, especially in non-dopamine neurons of the ventral tegmental area (VTA), leading to increased drug-seeking behavior [53]. This also suggests that GluA1 and NR1 are involved in SUD. NMDA receptors play a role in the development of SUD, but the role of NR1 in CPP development remains unclear [54]. Extracellular signal-regulated kinase (ERK), cAMP response element-binding protein (CREB) [50,51] and postsynaptic density protein (PSD-95) play a crucial role in SUD [55], but changes in these proteins were not observed in FVB mice when compared with wild-type mice. Specifically, we observed changes in the glutamate system in FVB mice. Studies on the glutamate system in the process of SUD training, extraction, renewal, and reinstatement, among others, have determined that glutamate plays different roles within different brain regions [56,57]. The role of the glutamate system in both SUD and normal learning ability systems remains to be explored.

There are several limitations of this study. Because fewer C57 mice showed a negative correlation between learning ability and SUD, an insufficient number of mice could not be screened for intervention experiments, such as brain region and circuit intervention. Therefore, this hypothesis was not further validated in C57 mice. However, we determined the hypothesis from C57 mice and applied them in the context of studies in FVB mice. In this study, the impaired learning and memory of FVB mice were not the sole reason for the high susceptibility to SUD, we will focus on other factors (such as genetic factors and brain circuits) that affect susceptibility to SUD in later research. In the future, we will also screen mice with strong learning abilities to study SUD susceptibility. Nonetheless, this experiment established a relationship between learning ability and SUD susceptibility, which provides a basis tool for the precise prevention and control of SUDs. Because the methods of testing learning ability are diverse and easy to obtain, SUD susceptibility screening through learning ability is simpler and easier when applied to the general population. A clear causal relationship between learning deficits in FVB mice and high CPP priming performance in this study has not been directly demonstrated, and this will also be the focus of our further research.

In summary, there is a relationship between poor learning ability and strong SUD susceptibility in human and animal models. This indicates that the lack of learning ability may be an indicator of high SUD susceptibility. Therefore, it may be a simple and practical method to identify high-risk groups in the normal population and provide tools for the precise prevention and control of SUD through future learning capabilities.

## Data Availability

Not applicable.
